# [^18^F]-Fluciclovine PET discrimination of recurrent intracranial metastatic disease from radiation necrosis

**DOI:** 10.1186/s13550-020-00739-6

**Published:** 2020-12-07

**Authors:** Ephraim E. Parent, Dhruv Patel, Jonathon A. Nye, Zhuo Li, Jeffrey J. Olson, David M. Schuster, Mark M. Goodman

**Affiliations:** 1 Department of Radiology, Mayo Clinic, Jacksonville, USA; 2 Department of Radiology and Imaging Sciences, Emory University School of Medicine, 1841 Clifton Rd. NE, 2nd Floor, Atlanta, GA 30329 USA; 3 Department of Statistics, Mayo Clinic, Jacksonville, USA; 4 Department of Neurosurgery, Emory University School of Medicine, Atlanta, GA USA

**Keywords:** ^18^F-fluciclovine, Brain metastasis, Radiation necrosis, Amino acid, PET

## Abstract

**Background:**

Stereotactic radiosurgery (SRS) is often the primary treatment modality for patients with intracranial metastatic disease. Despite advances in magnetic resonance imaging, including use of perfusion and diffusion sequences and molecular imaging, distinguishing radiation necrosis from progressive tumor remains a diagnostic and clinical challenge. We investigated the sensitivity and specificity of ^18^F-fluciclovine PET to accurately distinguish radiation necrosis from recurrent intracranial metastatic disease in patients who had previously undergone SRS.

**Methods:**

Fluciclovine PET imaging was performed in 8 patients with a total of 15 lesions that had previously undergone SRS and had subsequent MRI and clinical features suspicious for recurrent disease. The SUVmax of each lesion and the contralateral normal brain parenchyma were summated and evaluated at four different time points (5 min, 10 min, 30 min, and 55 min). Lesions were characterized as either recurrent disease (11 of 15 lesions) or radiation necrosis (4 of 15 lesions) and confirmed with histopathological correlation (7 lesions) or through serial MRI studies (8 lesions).

**Results:**

Time activity curve analysis found statistically greater radiotracer accumulation for all lesions, including radiation necrosis, when compared to contralateral normal brain. While the mean and median SUV_max_ for recurrent disease were statistically greater than those of radiation necrosis at all time points, the difference was more significant at the earlier time points (p = 0.004 at 5 min–0.025 at 55 min). Using a SUV_max_ threshold of ≥ 1.3, fluciclovine PET demonstrated a 100% accuracy in distinguishing recurrent disease from radiation necrosis up to 30 min after injection and an accuracy of 87% (sensitivity = 0.91, specificity = 0.75) at the last time point of 55 min. However, tumor-to-background ratios (TBR_max_) were not significantly different between recurrent disease and radiation necrosis at any time point due to variable levels of fluciclovine uptake in the background brain parenchyma.

**Conclusions:**

Fluciclovine PET may play an important role in distinguishing active intracranial metastatic lesions from radiation necrosis in patients previously treated with SRS but needs to be validated in larger studies.

## Background

Intracranial brain metastases from an extracranial primary lesion are seen in 24–45% of patients with known melanoma, lung, breast, and renal primary cancers [1]. Imaging plays a crucial role in management of metastatic brain lesions, and conventional magnetic resonance imaging (MRI) has been the standard of care for detection, treatment planning, and post-treatment evaluation of brain metastasis [2]. In patients previously treated with SRS, both tumor recurrence and radiation necrosis lead to clinical deterioration and can have a similar appearance on both anatomic imaging with MRI and computed tomography (CT), as well as with metabolic scans such as magnetic resonance spectroscopy and positron emission tomography (PET) [3, 4].

2-deoxy-2[^18^F]fluoro-D-glucose (FDG) PET has been shown to have limited ability to accurately identify recurrent intracranial metastatic disease after radiation therapy [5, 6]. Many tumors exhibit upregulated amino acid transporter expression and, as a result, have been shown to concentrate radiolabeled amino acids [7, 8]. Because normal brain parenchyma does not concentrate significant amino acid radiotracer, radiolabeled amino acids can provide a high signal-to-noise ratio and improve evaluation in the post-radiation setting where radiation necrosis may be confused with recurrent disease on MRI and clinical evaluation [9, 10].

Amino acid PET including L-methyl-[C-11]-methionine ([C-11]-MET) PET and 6-[F-18]-fluoro-L-dopa ([F-18]-FDOPA) PET has been shown to help differentiate active malignancy from radiation necrosis for intracranial metastasis [11–13]. Anti-1-amino-3-[F-18]-fluorocyclobutane-1-carboxylic acid, *anti*-3-[F-18] FACBC (fluciclovine), is a synthetic amino acid that has been shown to have high uptake in tumors and was approved by the US Food and Drug Administration (FDA) in May 2016 for the indication of biochemical evidence of recurrent prostate cancer [14]. It has also been shown to have increased uptake in brain glioma for which it has been granted orphan drug status [15–18]. Recently, it has also been reported to differentiate between high-grade and low-grade gliomas [15]. The purpose of this study was to investigate the ability of fluciclovine to discriminate recurrent intracranial metastatic disease from radiation necrosis in patients previously treated with SRS. We hypothesized that using simple semiquantitative PET parameters, such as maximum standardized uptake value (SUVmax), fluciclovine would be able distinguish recurrent metastatic disease from radiation necrosis in patients with known intracranial metastatic disease.

## Methods

### Patient population

#### Subject recruitment

Patients with biopsy-proven primary brain glioma or intracranial metastatic disease were recruited from 09/17/2000 to 11/18/2002. All procedures performed in studies involving human participants were in accordance with the ethical standards of the institutional and/or national research committee and with the 1964 Declaration of Helsinki and its later amendments or comparable ethical standards. The recruitment protocol was approved by the Institutional Review Board (IRB) and complied with the Health Insurance Portability and Accountability Act (HIPAA). The data were collected as part of a phase I trial for whole-body imaging under the IRB title ‘Imaging Analysis of Amino Acid Metabolism In Intracranial Tumors Using PET and ^18^F-FACBC (IRB#00101652)’ and submitted on 1/27/2002. This study is not listed on clinicaltrials.gov because when it was submitted, clinicaltrails.gov was only requiring NIH-funded projects be listed. After completion of a related fluciclovine PET study to evaluate gliomas [15], we have now analyzed the original dataset to focus on patients with suspected recurrent intracranial metastatic disease. Informed consent was obtained from all individual participants included in the study. The radiotracer was administered under FDA Investigational New Drug (IND) 72,437 and was synthesized either via automated synthesis [19] or the FASTlab Cassette System (GE Healthcare). Safety monitoring during the drug infusion was performed, and no adverse events were recorded.

For this analysis, patients were sub-selected under the inclusion criteria of intracranial metastatic disease previously treated with SRS, with a mean time between completion of SRS and fluciclovine PET of 10.5 months (range 1.2–18.3 months). In total, 25 patients were recruited and received at least one fluciclovine PET study post-histologic confirmation as required by the IRB. No patients had undergone fluciclovine PET studies prior to SRS for comparison. Patients were followed up with either excisional biopsy/partial resection or serial brain MRI examinations for up to 2 years or death. Patients that were lost to follow-up were not included in this analysis. The reference standard for disease progression was histologic proof when possible or progressive enlargement on serial MRI. A negative biopsy or stability/improvement on serial MRI without interval therapy was considered consistent with radiation necrosis. It is well known that histological analysis of radiation necrosis often finds three distinct patterns: viable tumor, radiation necrosis and most often a mix of necrosis and viable cells [20], with this final combination possibly confounding image interpretation. Unfortunately, due to the delay between tissue sampling and this study, the original tissue samples were not available for review and the negative or positive biopsy results are based on the original pathology report. Patients were excluded from analysis if they received SRS within 5 weeks prior to fluciclovine PET to exclude confounding inflammation from acute radiotherapy. This resulted in a total of 8 patients with 15 lesions included in this study (Table 1).

**Table 1:** Patient demographics

Age	52 years (39–86)	
Gender	4 Male	4 Female
Primary tumor	Patients	Lesions
Lung	4 (50%)	5 (33%)
Renal	1 (13%)	4 (27%)
Breast	2 (25%)	3 (20%)
Colon	1 (13%)	3 (20%)

### Image acquisition

All studies were collected on an ECAT 921 dedicated PET scanner in 3D mode consisting of 24 crystal rings spanning a field of view 16.2 cm resulting in 47 reconstructed image planes. Fluciclovine scans were acquired over 65 min in dynamic mode and started concurrently with injection of 357 ± 24 MBq of activity. The data were collected in sonogram mode and binned into four time points (2 × 5 min, 1 × 20 min, and 1 × 25 min). PET emission data were corrected for attenuation, randoms, and scatter and reconstructed with a filtered backprojection algorithm and Hanning filter (0.4 × Nyquist frequency) giving an in-plane resolution of 7.8 mm full width at half maximum (FWHM) and axial resolution of 6.2 mm FWHM. Data were transferred to a MIM workstation (MIM Software, OH) for further analysis.

### Selection of regions of interest (ROIs)

A board-certified radiologist using the Absolute Threshold Contouring Tool (MIM Software, OH, USA) drew ROIs over the tumors and background ROIs (i.e., contralateral brain and venous confluence) for all time points. Fluciclovine PET images were co-registered to T1 post-contrast MRI and fluid-attenuated inversion recovery (FLAIR) sequences. Tumor ROIs were defined by creating a spherical PET ROI to include the volume of tissue demonstrating contrast enhancement corresponding to known intracranial metastatic deposit. Within this PET ROI, the voxels with peak activity were used to derive a tumor maximum standardized uptake value (SUV_max_). A 15-mm spherical ROI was placed over the contralateral normal brain, including both gray and white matter as appropriate, to obtain a normal maximum standardized uptake value (SUV_max_normal_). Careful consideration when drawing ROIs over the tumor was used to exclude blood pool or adjacent choroid plexus which could falsely contribute to artifactually elevated SUV values.

### Semiquantitative PET metrics

SUV_max_ for each lesion and contralateral normal parenchyma was recorded at all time points. Tumor-to-background ratios for each lesion were calculated as TBR_max_ = (SUV_max_ tumor)/( SUV_max_normal_) at all imaged time points.

Estimating threshold values for classification of recurrent metastatic disease versus radiation necrosis.

The optimal threshold for differentiating radiation necrosis from recurrent disease utilizing tumor SUV_max_ was calculated using a receiver operator characteristic curve (ROC) for each lesion SUV_max_ measurements from 5 to 55 min post-injection. Sensitivity and specificity for identifying radiation necrosis are reported based on the optimal threshold. A similar approach using ROC curves was applied to each TBR_max_ dataset to distinguish recurrent metastatic disease versus radiation necrosis.

## Statistical analysis

For each time point, mean SUVmax and the standard deviation of SUVmax were calculated for normal brain parenchyma and brain lesions. Statistical significance between malignant lesions and radiation necrosis was determined using Wilcoxon rank sum test. All tests were two-sided with alpha level set at 0.05 for statistical significance. R3.6.1 was used for analysis.

## Results

### Subject demographics

Eight patients (4 male and 4 female) with intracranial malignancies previously treated with SRS and a mean age of 52 years (range 39y—86y) were included per the inclusion criteria (Table 1).

One patient had 4 lesions, another had 3 lesions, two patients had 2 lesions each, and the remaining four had 1 lesion each, resulting in a total of 15 distinct lesions being independently evaluated. Lung cancer was the most common primary malignancy with the highest number of patients (4 of 8) and metastatic lesions (5/15). Other primary malignancies included renal (1 patient/4 lesions), breast (2 patients/3 lesions), and colon (1 patient/3 lesions). All patients completed the fluciclovine PET scan after standard-of-care MRI demonstrated an enhancing lesion in an area previously treated with SRS, with mean time between completion of SRS and fluciclovine PET of 10.5 months (range 1.2 to 18.3 months). Histological confirmation via stereotactic biopsy/excisional biopsy was obtained for 7 lesions with the remaining 8 lesions classified with either progressive enhancement (recurrent tumor) or stable/decreasing enhancement (radiation necrosis) on subsequent standard-of-care MRI examinations. Based on their subsequent pathological and/or MRI findings, 11 lesions met criteria for recurrent disease and 4 lesions met criteria for radiation necrosis. Histological verification included 5 of 11 lesions (45%) with recurrent disease and 2 of 4 patients (50%) with radiation necrosis. PET imaging was performed an average of 11.1 months (range 1 months—18 months) after completion of SRS.

### Semiquantitative PET metrics and threshold values

ROC analysis was performed for each lesion and compared to contralateral normal brain parenchyma at four different time points: 5 min, 10 min, 30 min, and 55 min (Table 2). Each lesion, including those with radiation necrosis, demonstrated statistically greater radiotracer accumulation compared to normal brain parenchyma at each time point. The mean and median SUV_max_ for recurrent disease were similarly statistically greater than those of radiation necrosis at all four time points, and greater at the earlier time points (p = 0.004 at 5 min–0.025 at 55 min). Retrospective analysis provided an optimum SUV_max_ threshold of ≥ 1.3 to distinguish recurrent disease from radiation necrosis. Using this threshold, fluciclovine PET demonstrated a 100% accuracy at the 5, 10, and 30 min time points and an accuracy of 87% at the 55 min time point (sensitivity = 0.91, specificity = 0.75).

**Table 2:** SUVmax values for recurrent disease, radiation necrosis, and normal brain

	Recurrent disease (N = 11)	Tumor necrosis (N = 4)	Background (N = 15)	p value
5 m Lesion				0.004
Mean (SD)	1.9 (0.6)	0.8 (0.1)	0.6 (0.2)	
Median (range)	2.0 (1.1, 3.1)	0.8 (0.7, 1.0)	0.6 (0.4, 0.9)	
5 min TBR_max_				0.121
Mean (SD)	3.0 (1.3)	1.8 (0.2)		
Median (range)	2.6 (1.3, 5.3)	1.8 (1.6, 2.2)		
10 min Lesion				0.033
Mean (SD)	2.3 (1.2)	0.9 (0.2)	0.6 (0.2)	
Median (range)	2.0 (1.4, 5.4)	0.9 (0.7, 1.0)	0.6 (0.4, 1.0)	
10 min TBR_max_				0.129
Mean (SD)	3.4 (1.8)	1.9 (0.5)		
Median (range)	3.1 (1.5, 7.8)	1.8 (1.5, 2.6)		
30 min Lesion				0.042
Mean (SD)	2.3 (1.1)	1.1 (0.2)	2.0 (1.1)	
Median (range)	2.2 (1.4, 5.3)	1.1 (0.8, 1.3)	1.9 (0.8, 5.3)	
30 min TBR_max_				0.178
Mean (SD)	3.6 (1.9)	2.2 (0.6)		
Median (range)	3.2 (1.4, 7.7)	2.3 (1.5, 2.8)		
55 min Lesion				0.025
Mean (SD)	2.3 (0.9)	1.1 (0.3)	0.7 (0.2)	
Median (range)	2.3 (1.3, 4.4)	1.1 (0.8, 1.4)	0.6 (0.2, 1.2)	
55 min TBR_max_				0.304
Mean (SD)	3.3 (1.5)	2.5 (0.9)		
Median (range)	3.2 (1.4, 5.7)	2.5 (1.4, 3.5)		

^*^P value for each time point between mean SUVmax of malignant metastatic lesion and radiation necrosis

**Fig. 1:**
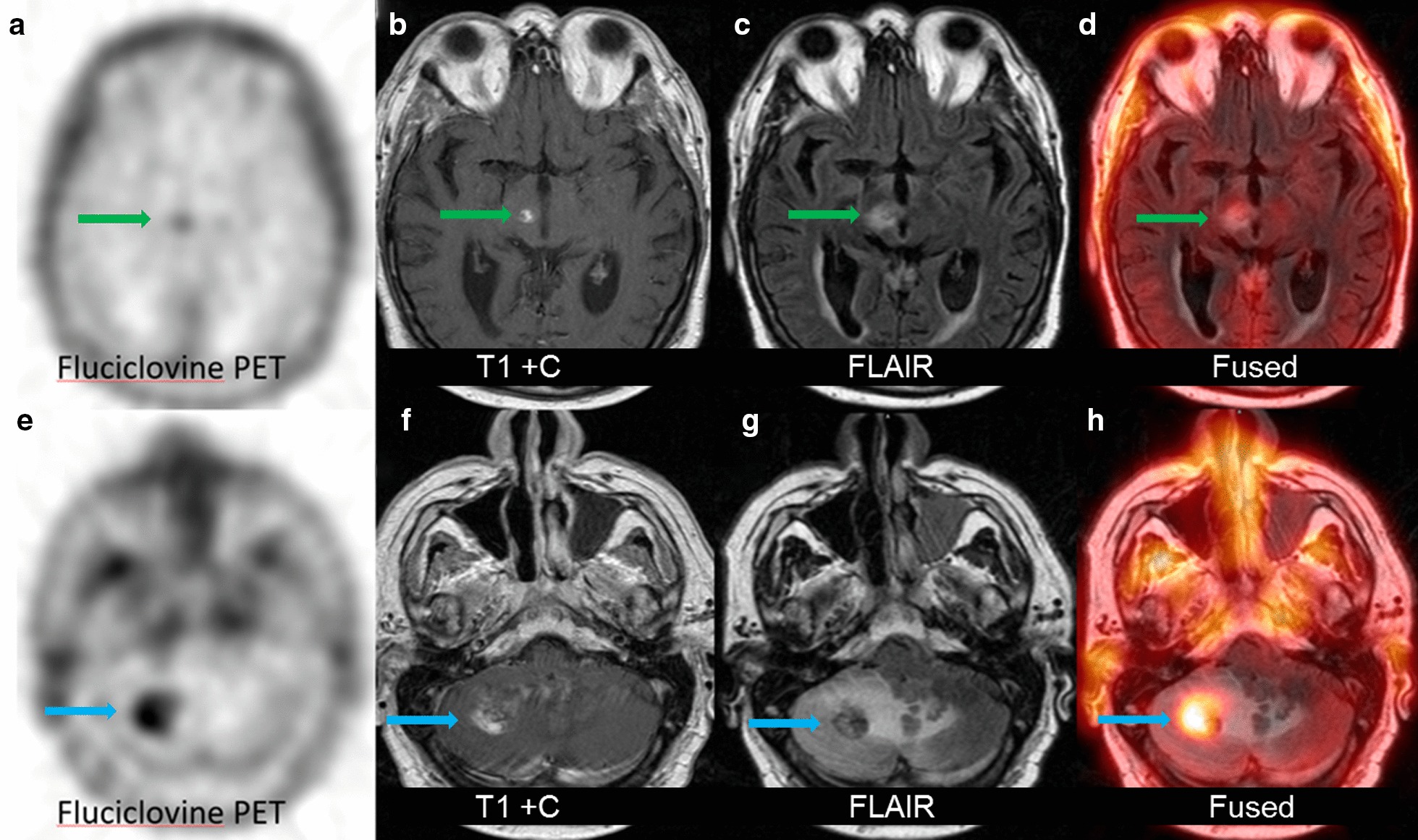
A 54-year-old patient with metastatic renal cell carcinoma and prior stereotactic radiosurgery. Follow-up MRI demonstrated progressively enhancing brain lesions suspicious for recurrent disease. Top panel demonstrates that a right thalamic lesion (green arrow) had low fluciclovine uptake (SUV_max_ of 1.0) as seen on transaxial PET (**a**), corresponding T1 + contrast (**b**), focal FLAIR hyperintensity (**c**), and fused FLAIR and PET (**d**). This lesion did not increase in size on follow-up MRI and was considered consistent with radiation necrosis. A right cerebellar lesion (blue arrow) in the same patient had high fluciclovine uptake (SUV_max_ of 5.3) on transaxial PET (**e**) and corresponding T1 + contrast (**f**) FLAIR hyperintensity (**g**) and fused FLAIR and PET (**h**). The right cerebellar lesion was found to be recurrent metastatic disease upon resection

**Fig. 2:**
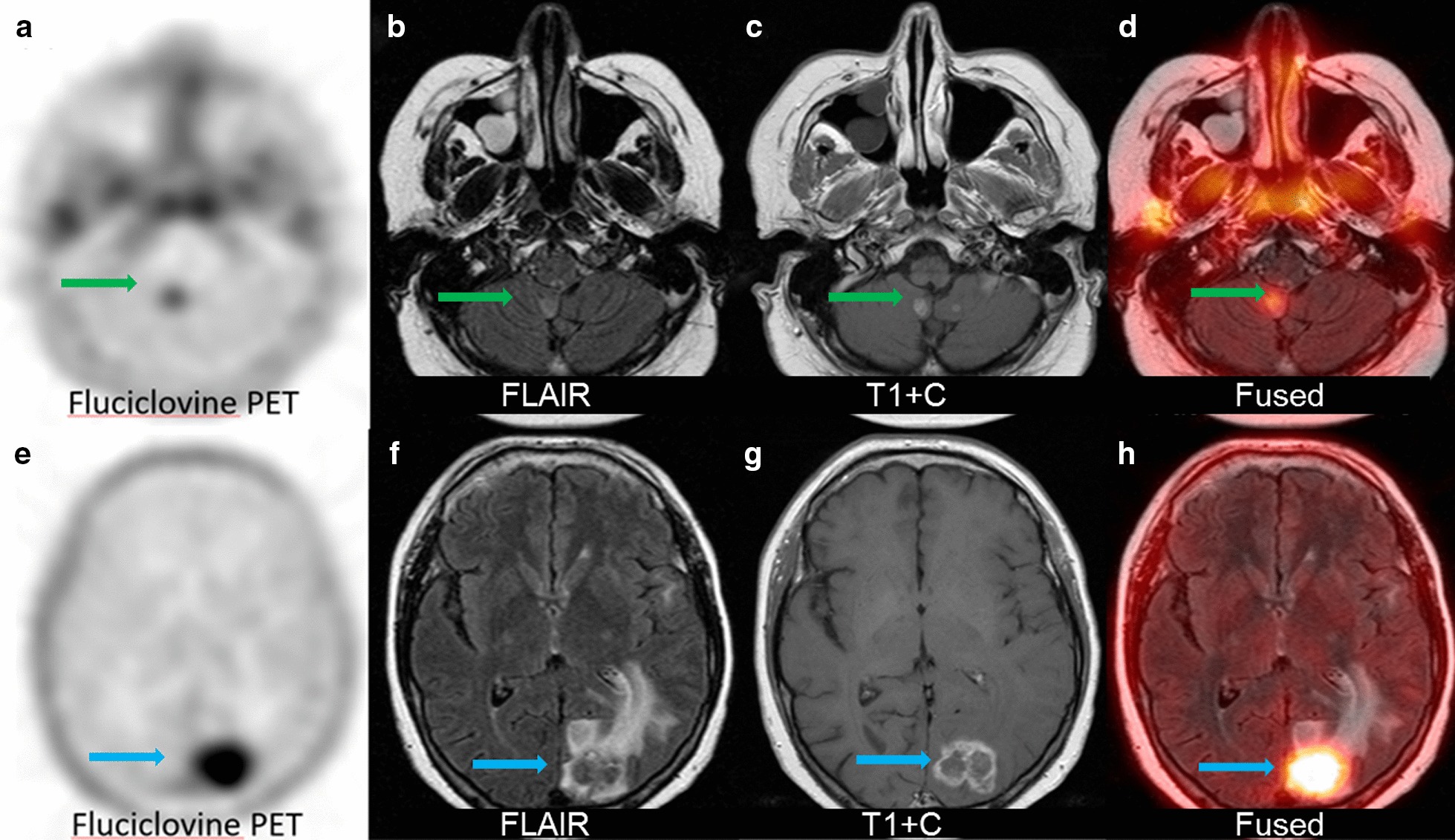
A 43-year-old patient with metastatic colon cancer with prior stereotactic radiosurgery with follow-up MRI demonstrating multiple enhancing brain lesions suspicious for recurrent disease. Top panel demonstrating a right cerebellar lesion (green arrow) with low fluciclovine uptake (SUV_max_ of 1.2) on transaxial PET (**a**) and corresponding focal FLAIR hyperintensity (**b**) T1 + contrast (**c**) and fused FLAIR and PET (**d**). This lesion did not increase in size on follow-up MRI and was consistent with radiation necrosis. A left occipital lesion (green arrow) in the same patient had high fluciclovine uptake (SUV_max_ of 2.5) on transaxial PET (**e**), hyperintense FLAIR (**f**), T1 + contrast enhancement (**g**), and fused FLAIR and PET (**h**). The left occipital lesion was found to be recurrent metastatic disease upon resection

In an attempt to normalize differences in physiologic vascular flow, the SUV_max_ of each lesion was normalized to the contralateral brain, TBR_max_ = (SUV_max_ tumor)/(SUV_max_normal_), and these values were compared between the two groups. However, TBR_max_ was not significantly different between recurrent disease and radiation necrosis at any time point in this analysis due to variable levels of fluciclovine uptake in the background brain parenchyma (Figs. 1, 2).

One patient with low fluciclovine uptake demonstrated progressive increase in enhancement on MRI at 2 and 4 months after fluciclovine PET and subsequently underwent surgical resection without intervening chemotherapy or radiation therapy, and final pathology was consistent with radiation necrosis. For the remainder of the lesions that underwent surgical resection, both pathology and follow-up MRI were consistent with each other.

## Discussion

Metastatic brain tumors are the most common brain tumor in adults, and the frequency of brain metastasis is increasing with up to 200,000 new cases every year [2]. External beam radiation therapy, in particular SRS, is considered part of first-line therapy for intracranial metastases [21]. The efficacy of SRS in patients with intracranial metastases has been shown to have control rates of 70–90% [22]. One of the most common problems of SRS for both primary brain gliomas and intracranial metastases is correctly identifying progressive reactive changes from radiation injury. Early true progression is difficult to distinguish from reactive changes (pseudoprogression) in the short term and irreversible injury (radiation necrosis) at latter time points [23]. Radiation necrosis is difficult to distinguish from tumor recurrence by both clinical presentation and imaging studies and can be seen in up to 25% of patients after SRS [24]. Both recurrent tumor and radiation necrosis demonstrate increased FLAIR signal and disruption of the blood brain barrier resulting in contrast enhancement [3, 13]. The ability to accurately identify true progression from therapy-related changes is critical as it enables appropriate therapeutic intervention. Even with MRI techniques such as perfusion [25] and spectroscopy [26], differentiation between radiation necrosis and active metastatic brain lesion is difficult and brain biopsy remains the gold standard [3].

FDG, while widely used, has discordant results in its ability to differentiate recurrent brain metastasis from radiation necrosis, possible due to different thresholds used in each study and elevated background brain parenchymal uptake [27]. Amino acid PET agents such as [F-18]-fluroethyltyrosine ([F-18]-FET) and [C-11]-MET [28, 29] have been used with some success as a means to differentiate progressive metastatic disease from radiation necrosis. [F-18]-FET TBR values have been shown to accurately identify recurrent metastases with metastatic uptake being significantly higher than that of radiation necrosis [30]. Additionally, dynamic FET PET imaging has been shown to improve accuracy in distinguishing recurrent disease with characteristic time–activity curves [31]. None of these most commonly used amino acid PET radiopharmaceuticals used for intracranial metastatic evaluation are yet FDA-approved and thus have limited application in research studies in the USA. Fluciclovine, on the other hand, is FDA-approved for evaluation of biochemically recurrent prostate cancer and has orphan drug status for evaluation of brain gliomas. Several other extra-prostatic malignancies including breast [32], renal [33], colon and lung (unpublished personal experience) have also been shown to have increased fluciclovine uptake. Our goal in this study was to evaluate the ability of fluciclovine to distinguish progressive metastatic lesions from radiation necrosis.

In this small sample set, all lesions, including both recurrent disease and radiation necrosis, demonstrated progressive post-contrast enhancement on prior standard-of-care MRI studies. There was overall good correlation between follow-up MRI findings and pathology results when available. It should be noted that there was a single lesion that was initially suggestive of recurrent disease on short-term follow-up with progressive increase in size and enhancement on subsequent follow-up MRI at 2 and 4 months. Conversely, there was low fluciclovine uptake in this lesion (SUV_max_ of 0.8 at 5 min increasing to 1.3 at 55 min) suggestive of radiation necrosis, and radiation necrosis was confirmed upon surgical resection and final pathology.

**Fig. 3:**
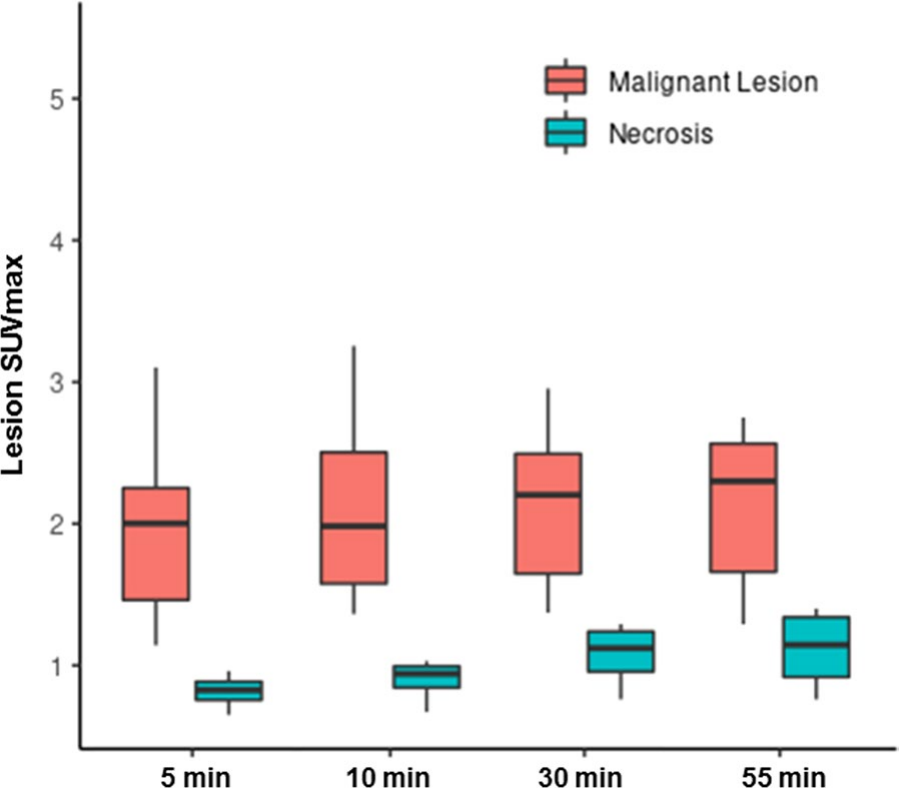
Box plot diagram of SUVmax values of recurrent disease and radiation necrosis

**Fig. 4:**
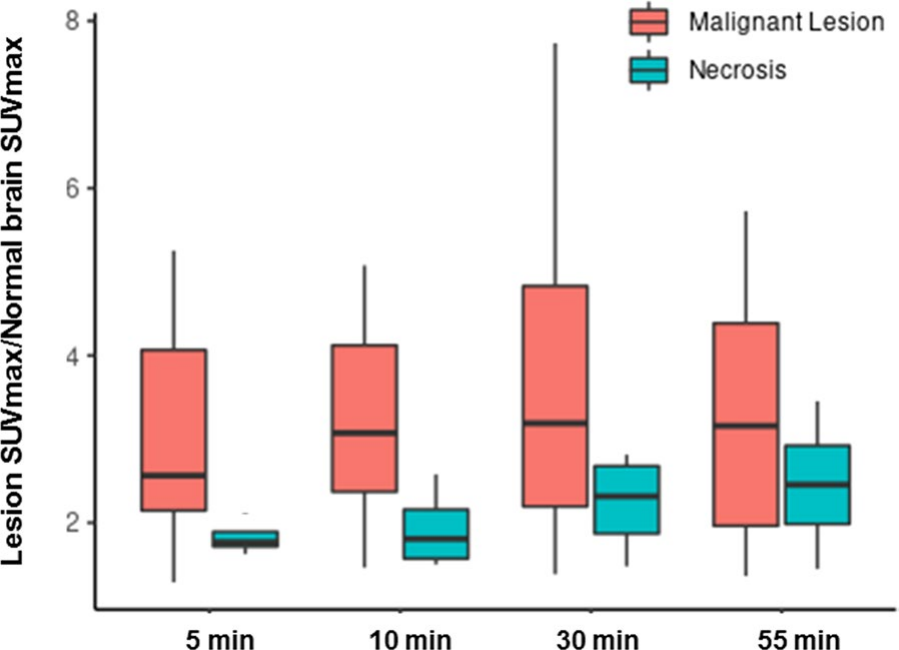
Box plot diagram of TBR_max_ = (SUV_max_ tumor)/( SUV_max_normal_) of recurrent disease and radiation necrosis

It is important to note that fluciclovine uptake in the recurrent disease was relatively stable over the 55 min of imaging (Fig. 3). Conversely, fluciclovine PET uptake in radiation necrosis showed mild progressively increased uptake for the duration of the uptake scan resulting in lower accuracy at the 55 min time point. These observations suggest that there may be differing time–activity curves between lesions with recurrent disease and radiation necrosis which may further help distinguish them from each other, although further investigation is needed (see supplemental material). Moreover, it appears that optimal timing of image acquisition to distinguish radiation necrosis from recurrent disease for fluciclovine is at early time points (up to 30 min) as progressive fluciclovine uptake in radiation necrosis lesions at later times points may confound discrimination. It is important to note that although fluciclovine uptake in radiation necrosis was lower compared to that of recurrent disease, it remained greater compared to contralateral normal brain parenchyma. This is possibly due to fluciclovine accumulation in inflammatory processes which while less than with FDG is still present [34]. Lastly, the overlap of fluciclovine uptake also likely reflects the heterogeneity of the treated lesions with coexistent viable tumor and radiation-related changes which are typically seen on histological examination [35] (Fig. 4).

There are several limitations to our study. First is the small patient population in both the recurrent disease and radiation necrosis groups, with a total of 8 patients having 15 lesions. Of these lesions, 11 met criteria for recurrent disease and only 4 were in radiation necrosis. Despite such a small number of patients and lesions, we were able to achieve statistical significance in fluciclovine uptake between two groups with an optimal SUV_max_ threshold of ≥ 1.3. However, it is important to note that this threshold determination is considered preliminary at best due to the small sample size. As far as we can determine, there are no other published studies using fluciclovine PET as a metric to distinguish radiation necrosis from recurrent intracranial metastatic disease and thus meta-analysis against our results is not possible. A larger fluciclovine PET-MRI study is currently accruing patients at our institution building upon these preliminary data and comparing more complex PET parameters with advanced MRI techniques to distinguish radiation necrosis from recurrent disease. Secondly, pathological confirmation was not available for all of the patients and lesions were categorized based on follow-up MRI findings which is not ideal. However, in all instances in which histological verification was available, fluciclovine findings were consistent with pathology (Fig. 2), even when MRI suggested otherwise. An addition limitation is that this study included intra-cranial metastatic disease from four different primaries and no patients had fluciclovine PET prior to SRS to demonstrate fluciclovine uptake in areas of viable disease. Moreover, this study was not powered for evaluation of intracranial metastasis from any one individual primary malignancy. Finally, it is not known if there is an optimal temporal point after radiation therapy to discriminate between the two etiologies.

As mentioned previously, further investigation with larger datasets is needed to confirm these preliminary findings and to further establish optimal PET imaging parameters. Specific questions that are being evaluated include optimal timing of fluciclovine PET for evaluation of brain metastasis in post-radiation patients and evaluation of more complete fluciclovine PET parameters (e.g., SUVmean, SUVpeak, TBR_max/mean_) and comparison with advanced MRI techniques (e.g., spectroscopy and perfusion). The observed difference in background brain fluciclovine uptake between patients with recurrent disease and radiation necrosis is unable to be adequately explained and is believed to be an artifact from the small sample size and will be further evaluated on a planned study with a larger sample size. If fluciclovine PET is found to have a high accuracy in distinguishing recurrent disease from radiation necrosis, this may help guide biopsy and obviate the current need for serial MRI evaluation after treatment, saving both time and money.

## Conclusions

Accurate discrimination between recurrent intracranial metastatic disease and radiation necrosis remains a radiographic and clinical dilemma in patients that have previously undergone SRS. Visual and semiquantitative analysis of fluciclovine PET is able to correctly identify radiation necrosis from recurrent disease and background brain parenchyma. The simple semiquantitative metric of SUV_max_ afforded a threshold of ≥ 1.3 to discriminate between recurrent disease and radiation necrosis. The SUV_max_ difference between radiation necrosis and recurrent disease was more pronounced at the earlier time points as radiation necrosis was found to slowly increase over time, whereas the fluciclovine uptake of recurrent disease remained relatively flat over 55 min after injection. While these results need to be evaluated in a larger sample size, fluciclovine PET may play an important role in distinguishing metabolically active intracranial metastatic lesions from radiation necrosis in patients previously treated with SRS.

## Supplementary information

**Additional file 1.** Time Activity Curve analysis of recurrent disease and radiation necrosis.

## Data Availability

Please contact author for data requests.

## Abbreviations

FDG: 2-Deoxy-2[^18^F]fluoro-d-glucose
[C-11]-MET: L-methyl-[C-11]-methionine
[F-18] FDOPA: 6-[F-18]-fluoro-l-dopa
FDA: US Food and Drug Administration
IRB: Institutional Review Board
HIPAA: Health Insurance Portability and Accountability Act
IND: Investigational New Drug
FWHM: Full width at half maximum
FLAIR: Fluid-attenuated inversion recovery
[F-18]-FET: [F-18]-fluroethyltyrosine
TBR: Tumor-to-background ratio
