# Supplementary material for: [18F]-Fluciclovine PET discrimination of recurrent intracranial metastatic disease from radiation necrosis
Source: EJNMMI Res. 2020 Dec 7;10:148. doi: 10.1186/s13550-020-00739-6 (PMC7721921; doi:10.1186/s13550-020-00739-6)
Supplement: Supplementary file 1 — Additional file 1. Time Activity Curve analysis of recurrent disease and radiation necrosis. [file 13550_2020_739_MOESM1_ESM.docx]

Supplemental Material

Time activity curves (TACs) of averaged lesion SUVmax and contralateral parenchymal SUVmax are shown beginning at 5 min (after completion of the 4 min infusion) to focus on equilibrium kinetics (supplemental figure 1). The reason for the slow 4 min infusion was based on the initial desire to broaden the input function so that one could accurately measure fluciclovine levels temporally when setting up multiple frame reconstruction. Future studies are currently being performed with bolus injection of fluciclovine to better evaluate the early kinetic uptake profiles. Dynamic analysis of recurrent disease implies a flattening/decrease of the TACs suggestive of a fluciclovine influx/efflux equilibrium starting at approximately 10 min post-injection; however, this needs to be validated due to our small sample size. Dynamic analysis of lesions found to be radiation necrosis implies a gradual increase in uptake, but again this needs to be evaluated due to the small sample size. Both sets of normal brain demonstrated stable low uptake throughout the evaluated period.

**Supplemental Figure 1**


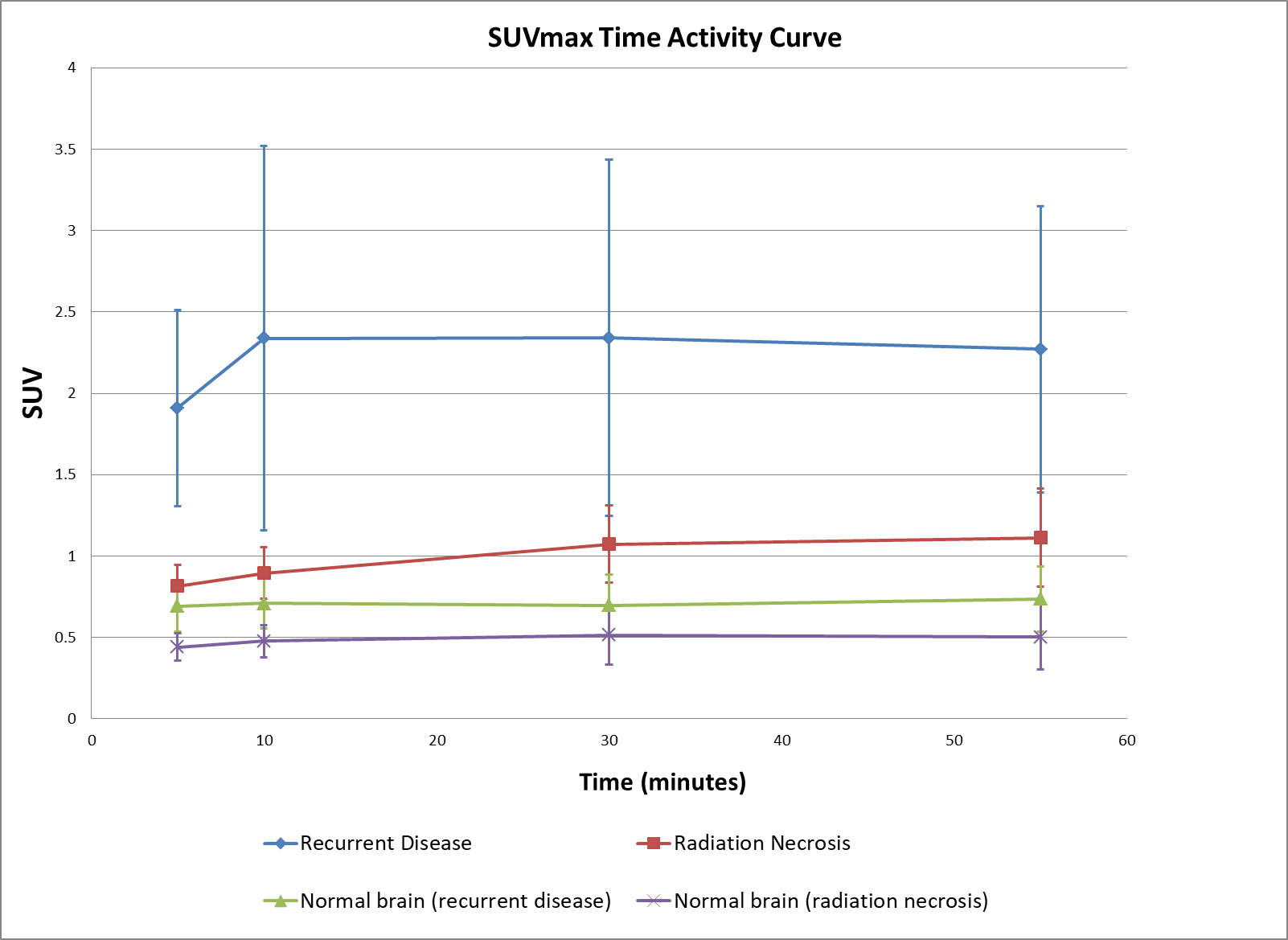


Time activity curves of recurrent disease (blue) and radiation necrosis SUVmax (red) show equilibrium kinetics starting 10 min post-injection. Statistically significant differences are noted between recurrent disease and radiation necrosis, and normal brain parenchyma (green and purple).

Each suspicious lesion was compared to the contralateral normal (TBR_max)_ but no significantly different between recurrent disease and radiation necrosis at any time point in this analysis due to variable levels of fluciclovine uptake in the background brain parenchyma between the two groups. It is uncertain as to why the background parenchyma between the radiation necrosis group and recurrent disease group were different. It is believed that the difference is due to statistical fluctuation due to the small sample size and this pattern is currently being explored in a separate study.
